# Genome-wide identification of heat shock factors and heat shock proteins in response to UV and high intensity light stress in lettuce

**DOI:** 10.1186/s12870-021-02959-x

**Published:** 2021-04-17

**Authors:** Taehoon Kim, Shafina Samraj, Juan Jiménez, Celina Gómez, Tie Liu, Kevin Begcy

**Affiliations:** 1 University of Florida, Environmental Horticulture Department, Gainesville, Florida, 32611 USA; 2 University of Florida, Horticultural Science Department, Gainesville, Florida, 32611 USA

**Keywords:** Lettuce, Heat shock factors, Heat shock proteins, Gene duplication, Light conditions

## Abstract

**Background:**

Heat shock factors (Hsfs) and Heat shock proteins (Hsps) belong to an essential group of molecular regulators involved in controlling cellular processes under normal and stress conditions. The role of Hsfs and Hsps is well known in model plant species under diverse stress conditions. While plants Hsfs are vital components of the signal transduction response to maintain cellular homeostasis, Hsps function as chaperones helping to maintain folding of damaged and newly formed proteins during stress conditions. In lettuce (*Lactuca sativa*), a highly consumed vegetable crop grown in the field and in hydroponic systems, the role of these gene families in response to artificial light is not well characterized.

**Results:**

Using a genome-wide analysis approach, we identified 32 Hsfs and 22 small heat shock proteins (LsHsps) in lettuce, some of which do not have orthologs in Arabidopsis, poplar, and rice. LsHsp60s, LsHsp90s, and LsHsp100s are highly conserved among dicot and monocot species. Surprisingly, LsHsp70s have three times more members than Arabidopsis and two times more than rice. Interestingly, the lettuce genome triplication did not contribute to the increased number of LsHsp70s genes. The large number of LsHsp70s was the result of genome tandem duplication. Chromosomal distribution analysis shows larger tandem repeats of LsHsp70s genes in Chr1, Chr7, Chr8, and Chr9. At the transcriptional level, some genes of the LsHsfs, LsHsps, LsHsp60s, and LsHsp70s families were highly responsive to UV and high intensity light stress, in contrast to LsHsp90s and LsHsp100s which did not respond to a light stimulus.

**Conclusions:**

Our genome-wide analysis provides a detailed identification of Hsfs and Hsps in lettuce. Chromosomal location and syntenic region analysis together with our transcriptional analysis under different light conditions provide candidate genes for breeding programs aiming to produce lettuce varieties able to grow healthy under hydroponic systems that use artificial light.

**Supplementary Information:**

The online version contains supplementary material available at 10.1186/s12870-021-02959-x.

## Background

Increasing human population, climate change conditions, decrease in water availability, and pressure of pathogens and insects have influenced the way we grow crops. Hydroponics is a viable alternative to crop production that addresses many of these issues [1]. Vegetables and fruits are the most commonly grown hydroponic crops. Within these groups, lettuce (*Lactuca sativa*), an important vegetable crop with a diploid genome (2n = 2x = 18 chromosomes) [2], is one of the most common vegetables produced in hydroponic systems [1, 3, 4]. Some of the production and health benefits of lettuce include a short production cycle, small size as well as its rich fiber, vitamins, minerals, and phytochemicals content [5].

A large fraction of lettuce production is grown hydroponically and indoors, relying on the supply of a nutrient solution and artificial light. The intensity and properties of light are critical factors that regulate photosynthesis and plant growth. Optimization of nutrients and light throughout the production cycle can provide better growing conditions to indoor hydroponic systems [3, 4]. However, the constant supply of high-energy radiation in the form of UV or high intensity light can negatively alter not only plant growth and development [6, 7], but also the transcriptional pattern of gene expression [8, 9]. Under these conditions, the expression of stress responsive genes is critical to ensure plant fitness and normal development. Two gene families are known to be in the front line of stress responses in tackling adverse conditions: heat shock factors (Hsfs) and heat shock proteins (Hsps). Hsfs and Hsps are involved in stress response mechanisms that allow plants to control folding, accumulation, and degradation of proteins.

Heat shock factors are transcriptional activators that regulate gene expression of their target genes. Plants Hsfs regulate core components not only of the heat stress response but also of many other environmental stresses by modulating the gene expression dynamics of a large group of genes involved in maintaining cellular homeostasis [10, 11]. Among other genes, Hsfs control the transcriptional activation and accumulation of Hsps which function as chaperones helping to maintain folding of damaged and newly formed proteins during development and stress conditions [11, 12]. In Arabidopsis, more than two hundred genes are transcriptionally controlled by Hsfs in response to heat stress, indicating the vast control of this transcription factor family during stress conditions [13]. Hsfs have a well-conserved basic structure which has allowed their classification into three major classes: Hsfs type A, B, and C [11]. Hsfs type A is the largest group of Hsfs in most species including Arabidopsis (*Arabidopsis thaliana*), rice (*Oryza sativa*) and poplar (*Populus trichocarpa*) [12, 14, 15]. Hsfs type A are required for the early response of *Arabidopsis* to excess light [16] and heat stress [13]. While Hsfs type A are capable of transcriptional activation, Hsfs type B act as co-activators or repressors of gene expression [17–19]. For instance, Hsfs type B contribute to salt tolerance by promoting flavonoid biosynthesis in soybean [19], and inhibit transcription in Arabidopsis [17, 18]. In contrast, even though it is believed that members of the Hsfs type C do not have activator function [20, 21], *HsfC1b* was shown to regulate salt tolerance and development by mediating ABA response in rice [22]. Plant heat shock proteins are more abundant than Hsfs and are classified into five major subfamilies based on their molecular weight: small Hsps (sHsps), Hsp60s, Hsp70s, Hsp90s, and Hsp100s. Hsps are responsible for a variety of molecular functions including folding, assembly, stabilization, refolding, translocation, and degradation of proteins under normal developmental conditions as well as under stress conditions. Members of Hsps have been described in response to heat, cold, drought, salinity, and light stress in many monocot and dicot species [12, 14, 15, 23–25].

Most studies investigating lettuce in response to light have been focused on the effects of high-energy radiation with ultraviolet (UV) and high intensity light wavebands on crop quality [3] and yield [4]. However, the molecular responses of Hsfs and Hsps to different light treatments have not been characterized yet. Here, we describe a genome-wide analysis of Hsfs and Hsps genes under diverse light conditions. A comprehensive description of subclasses of Hsfs, sHsps, Hsp60s, Hsp70s, Hsp90s and Hsp100s has been generated providing novel candidate genes for lettuce breeding programs.

## Results

### Identification and properties of lettuce heat shock transcription factors

**Fig. 1:**
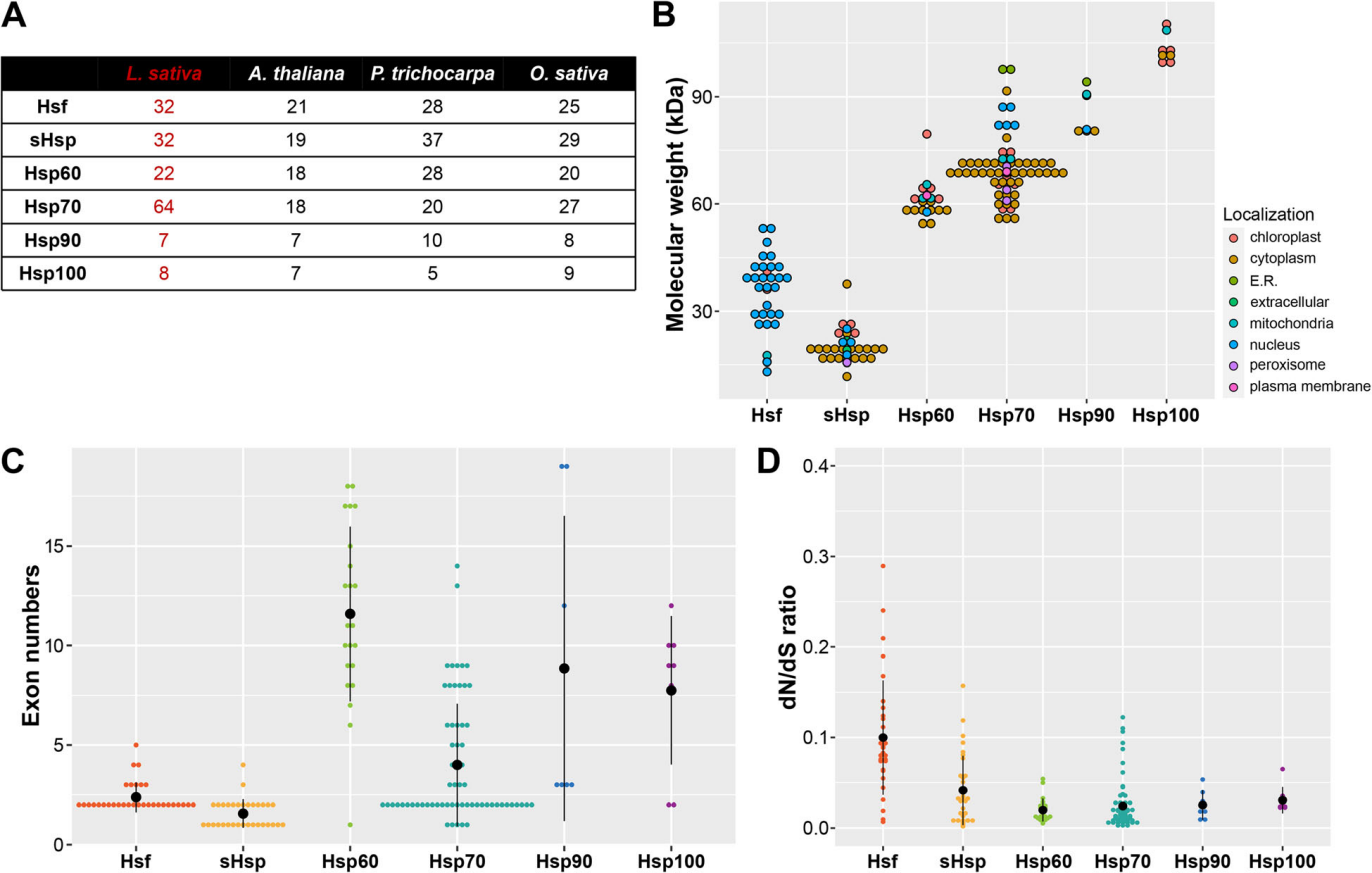
General gene structure features of lettuce Hsfs and Hsps. **a** Numbers of Hsf and Hsp genes in lettuce, Arabidopsis, poplar and rice. **b** Molecular weight analysis of LsHsfs and LsHsps. **c** Exon number distribution of the lettuce Hsfs and Hsps. **d** Evaluation of positive selection of LsHsfs and LsHsps families based on the dN/dS ratio analysis

Based on the recent sequencing of the lettuce genome [2], we identified LsHsfs family members using OrthoFinder [26] coupled with an automated local BLASTP search with the previously described Arabidopsis [12], rice [14]*,* and poplar [15] Hsfs as query sequences followed by manual curation. Previous Hsfs genome-wide studies have identified 21 AtHsfs, 28 OsHsfs, and 25 PtHsfs. Our automated search yielded 32 members of the LsHsfs family (Fig. 1a). Considering that the sequenced lettuce genome is a diploid plant with an estimated genome size of 2.5 Gb, it is interesting to note that lettuce carries a higher number of Hsfs compared to rice and poplar.

To further characterize LsHsfs, we collected their physiochemical features including chromosomal coordinates, molecular weight (MW), theoretical isoelectric point (pI), instability index, aliphatic index, hydropathicity, and predicted subcellular localization (Additional file 1: Table S1). Members of the LsHsfs family showed largely variable MWs, ranging from approximately 13 kDa to 54 kDa (Fig. 1b). Consistent with their putative function as transcription factors, most of the LsHsfs were predicted to be localized in the nucleus; however, LsHsfA1b and LsHsfA8 showed chloroplast signal peptides (Fig. 1b; Additional file 1: Table S1). It will be interesting to investigate whether both genes could play a dual function in the nucleus and the chloroplast. Another well conserved genome feature within the LsHsfs family was the number of exons, showing between 2 to 5 exons (Fig. 1c).

Because our analyses showed an increase in the number of LsHsfs, we decided to quantify the ratio of substitution rates at non-synonymous and synonymous sites in both LsHsfs and LsHsp to explore the evolutionary pressures on proteins during lettuce whole-genome triplication [2]. The largest dN/dS ratio was observed in LsHsfs (0.1); however, the hallmark signature of positive selection is accepted to be dN/dS > 1 [27]. All the LsHsps families showed dN/dS ratio of 0.5 or below (Fig. 1d). In general, no evidence of positive selection was found on any other member of the LsHsfs and the different LsHsps families based on the dN/dS ratio analysis (Fig. 1d; Additional file 2: Table S2).

### Classification, gene structure, and phylogenetic analysis of lettuce Hsfs

**Fig. 2:**
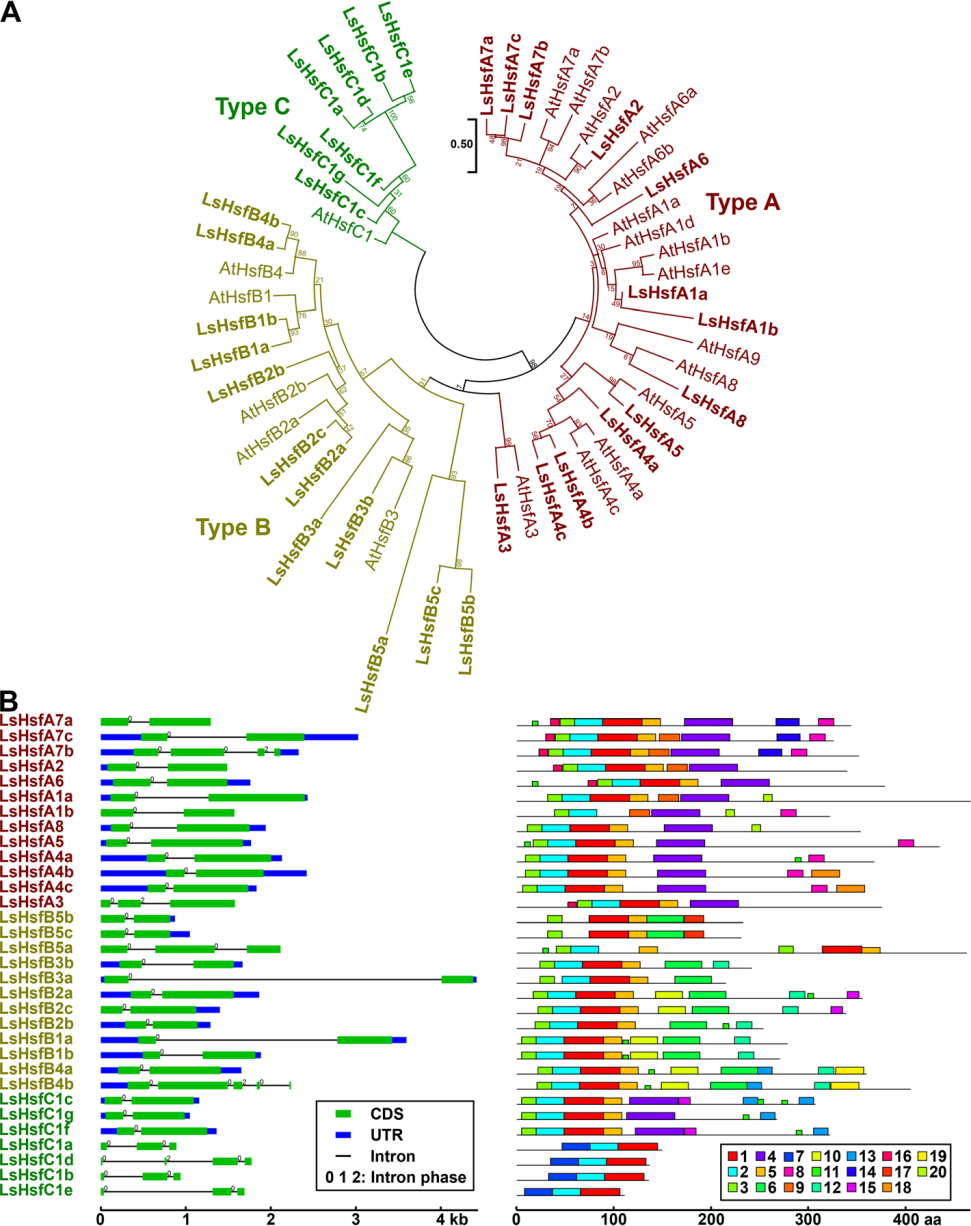
Phylogenetic analysis, exon-intron structure, and motif distribution of lettuce *heat shock transcription factor* (*LsHsf*) gene family. **a** Phylogenetic relationship of Hsf proteins of lettuce and Arabidopsis. *LsHsf* and *AtHsf* members in each subfamily (Type A, Type B, and Type C) are represented in different colors. **b** Gene structure and motif composition analysis of *LsHsf* genes with blue boxes, green boxes, and black lines indicate untranslated region (UTR), coding sequence (CDS), and intron, respectively. The numbers on each intron represent the intron phase (0, 1, or 2). Phylogenetic analysis was generated by the Maximum Likelihood method. Numbers at the nodes represent percent of bootstrap values based on 500 replications. Detailed information of all motifs is shown in Additional file 4: Table S4

Since Arabidopsis Hsfs are well characterized, we used its Hsfs proteins to generate orthogroups between Arabidopsis and lettuce using OrthoFinder (Additional file 3: Table S3). LsHsfs were classified in three main groups (Fig. 2a): type A (13 genes), type B (12 genes) and type C (7 genes). To further assess the evolutionary relationship of the Hsfs, a phylogenetic tree was constructed based on the full-length amino acid sequences from both lettuce and Arabidopsis (Fig. 2a). While most members of the type A subfamily in Arabidopsis had a similar number of orthologs lettuce genes, we found only two members (LsHsfA1a and LsHsfA1b) orthologs with four Arabidopsis Hsf genes in the subgroup A1. LsHsfs Type B and C subfamilies showed a larger number of new members compared to Arabidopsis. Interestingly, we identified a new lettuce subgroup that is not present in Arabidopsis, that we named LsHsfB5. It has three members, two of them belonging to the same orthogroup (*LsHsfB5b* and *LsHsfB5c*) and the additional member, *LsHsfB5a* forming an orthogroup by itself. Remarkably, we also identified a large number of LsHsf Type C (seven genes) compared to Arabidopsis (one gene), which mainly contributed to the overall increase in number of the LsHsfs family (Fig. 2a). The gene structure of the LsHsfs was highly conserved; however, four members of the LsHsfs type C (LsHsfC1a, LsHsfC1b, LsHsfC1d, LsHsfC1e) and three of the LsHsfs type B (LsHsfB1a, LsHsfB1b, LsHsfB1c) family displayed different gene structures and variable motif distributions (Fig. 2b).

### Lettuce small heat shock proteins (LssHsps)

**Fig. 3:**
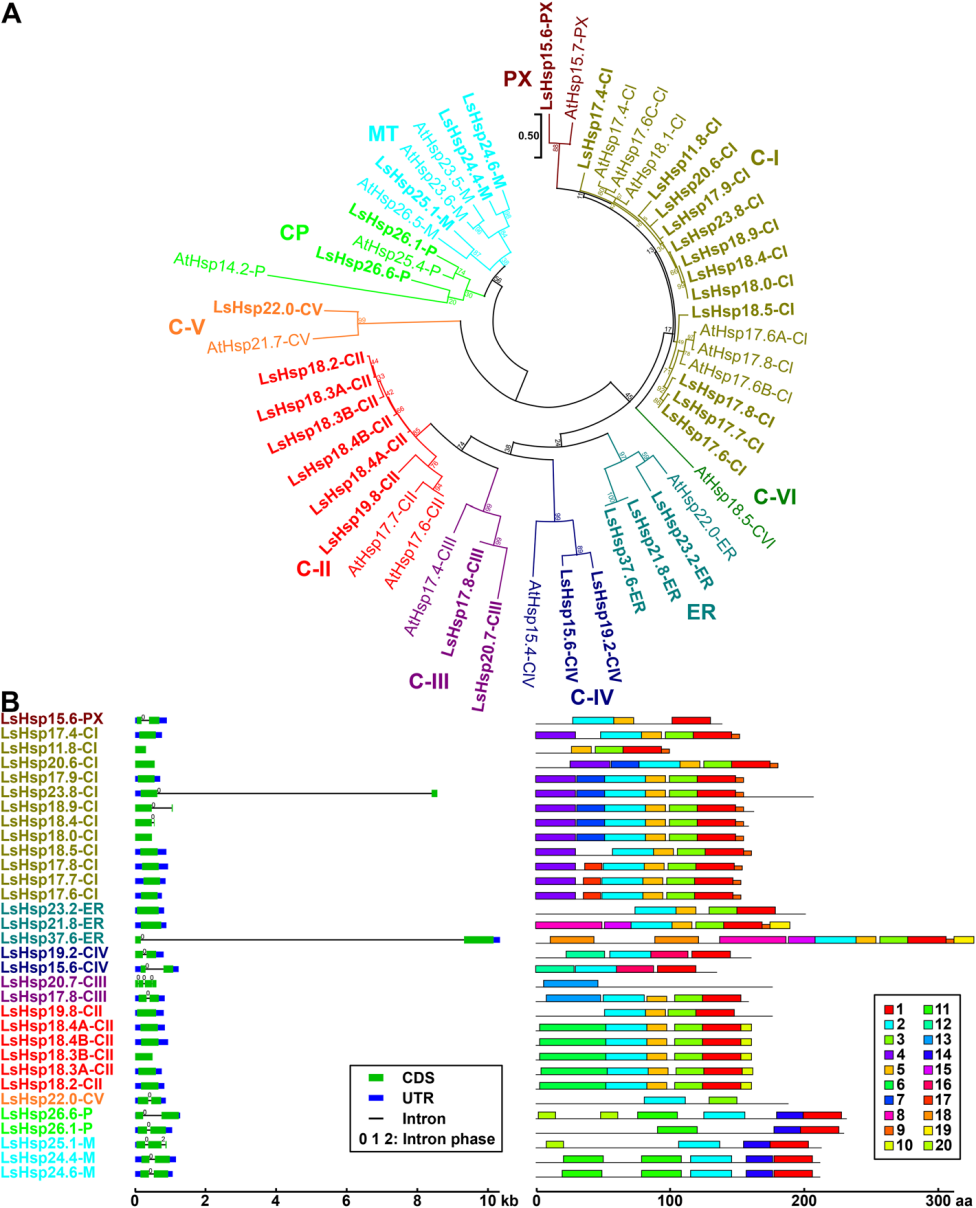
Identification, phylogenetic and gene structure analysis of lettuce *small heat shock protein* (*LssHsp*) gene family. **a** Phylogenetic relationship of LssHsp proteins and *A. thaliana*. LssHsp proteins are marked in bold. LssHsp and AtsHsp members in each subfamily (C-I, C-II, C-III, C-IV, C-V, C-VI, ER, MT, CP, and PX) are represented in different colors. Classification of *LssHsp* genes were assigned according to their molecular weight and subcellular localization (Additional file 1: Table S1). **b** Gene structure and motif composition analysis of *LssHsp* genes. Blue boxes, green boxes, and black lines indicate untranslated region (UTR), coding sequence (CDS), and intron, respectively. The numbers on each intron represent the intron phase (0, 1, or 2). Phylogenetic analysis was generated by the Maximum Likelihood method. Numbers at the nodes represent percent of bootstrap values based on 500 replications. Detailed information of all motifs is shown in Additional file 4: Table S4

We identified and classified lettuce small heat shock proteins (LssHsps) into nine subfamilies (Fig. 3a). Each subfamily was named based on their predicted MW and subcellular localization (Fig. 1b). Physiochemical features and predicted subcellular localization were also characterized (Additional file 1: Table S1). Five lettuce sHsp subfamilies are cytosol-localized (C-I, C-II, C-III, C-IV, and C-V). The other four subfamilies are predicted to be expressed in the endoplasmic reticulum (ER), peroxisome (PX), chloroplast (CP), and mitochondria (MT) (Fig. 3a). We found a large number of members belonging to the C-I (12 genes) and C-II subfamilies (6 genes) in the lettuce genome compared to only six C-I and two C-II genes in the Arabidopsis genome (Fig. 3a). Interestingly, we did not find LsHsp-CVI members based on their homology with Arabidopsis (Fig. 3a). Remarkably, the large majority of LssHsps have a single exon (Fig. 3b); nonetheless, the members of the LssHsp-PX, LssHsp-P, and LssHsp-M subclades have two or three exons. At the gene structure level, the exon/intron structures of LssHsps were relatively uniform except for LsHsp23.8-CI and LsHsp37.6-ER which contain long introns spanning 7.77 kb and 9.16 kb, respectively (Fig. 3b). In addition, even though each subfamily of LssHsps has some degree of conserved motifs, there are members showing variation in motifs. Remarkably, LssHsps subfamilies predicted to be expressed in the cytosol showed distinctive motifs (Motif 4) which could be an indication of their potential function in the plant cell (Fig. 3b).

### LsHsp60, a well conserved gene family

**Fig. 4:**
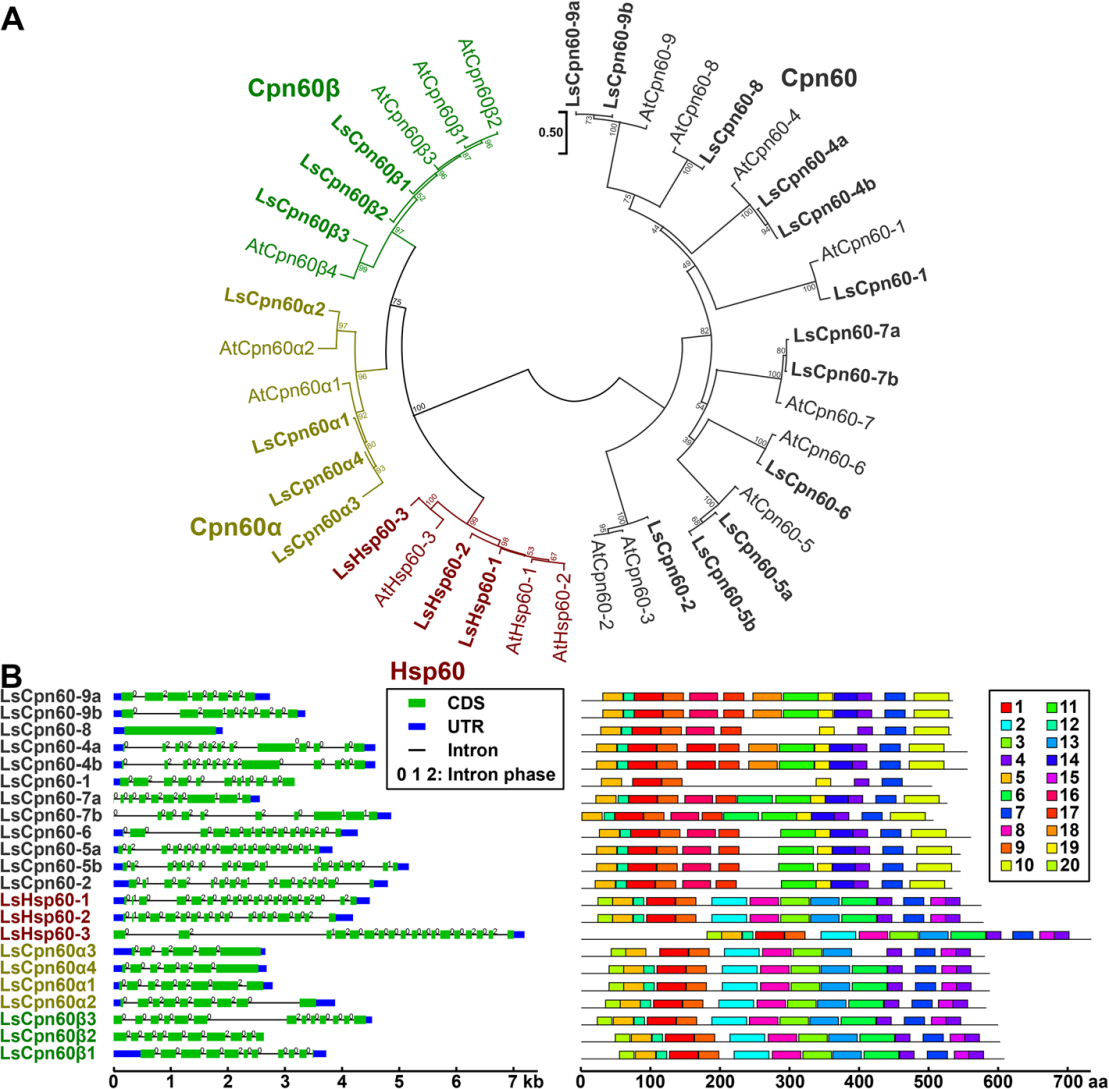
Classification, evolutionary relationship, gene structure and motif distribution of lettuce *heat shock protein 60* (*LsHsp60*) gene family**. a** Phylogenetic relationship of Hsp60 proteins of *L. sativa* and *A. thaliana*. LsHsp60 proteins are marked in bold. *LsHsp60* and *AtHsp60* members in each subfamily (Cpn60, Cpn60α, Cpn60β, and Hsp60) are represented in different colors. Nomenclature of *LsHsp60* genes was assigned according to the closest *AtHsp60* genes. **b** Gene structure and motif composition analysis of *LsHsp60* genes. Blue boxes, green boxes, and black lines indicate untranslated region (UTR), coding sequence (CDS), and intron, respectively. The numbers on each intron represent the intron phase (0, 1, or 2). Phylogenetic analysis was generated by the Maximum Likelihood method. Numbers at the nodes represent percent of bootstrap values based on 500 replications. Detailed information of all motifs is shown in Additional file 4: Table S4

Based on the phylogenetic relationships of the lettuce Hsp60 family with Arabidopsis Hsp60s, we classified LsHsp60 family into four subclasses: Cpn60 which contains 12 cytoplasmic genes; Cpn60α, having four chloroplastic members; Cpn60β, containing three chloroplastic members; and the Hsp60 subclass consisting of three mitochondrial genes (Fig. 4a). In general, Hsp60 genes are well conserved between Arabidopsis and lettuce given that each Arabidopsis Hsp60 member has at least one ortholog gene in lettuce. In addition, there are no Hsp60 lettuce-specific clade (Fig. 4a). At the gene structure level, the LsHsp60 has a well conserved motif structure; however, the number of exons varies across the different subfamilies (Fig. 4b). A physiochemical characterization of LsHsp60 was also conducted and can be found in Additional file 1: Table S1.

Another interesting feature of the LsHsp60 family is its variability in the intron phase. Based on the disruption of the last codon of an exon, introns have been divided into three types. The first is called phase 0, in which introns do not disrupt a codon. The second is called phase 1, in which introns disrupt a codon between the first and second bases. Finally, phase 2, where introns disrupt a codon between the second and third bases [28]. Unlike LsHsfs and the other LsHsps families, every LsHsp60 gene has at least one phase 0 and phase 2 intron, with the only exception of LsCpn60–8 which does not have any introns (Fig. 4b).

### Tandem gene duplication of lettuce Hsp70s

**Fig. 5:**
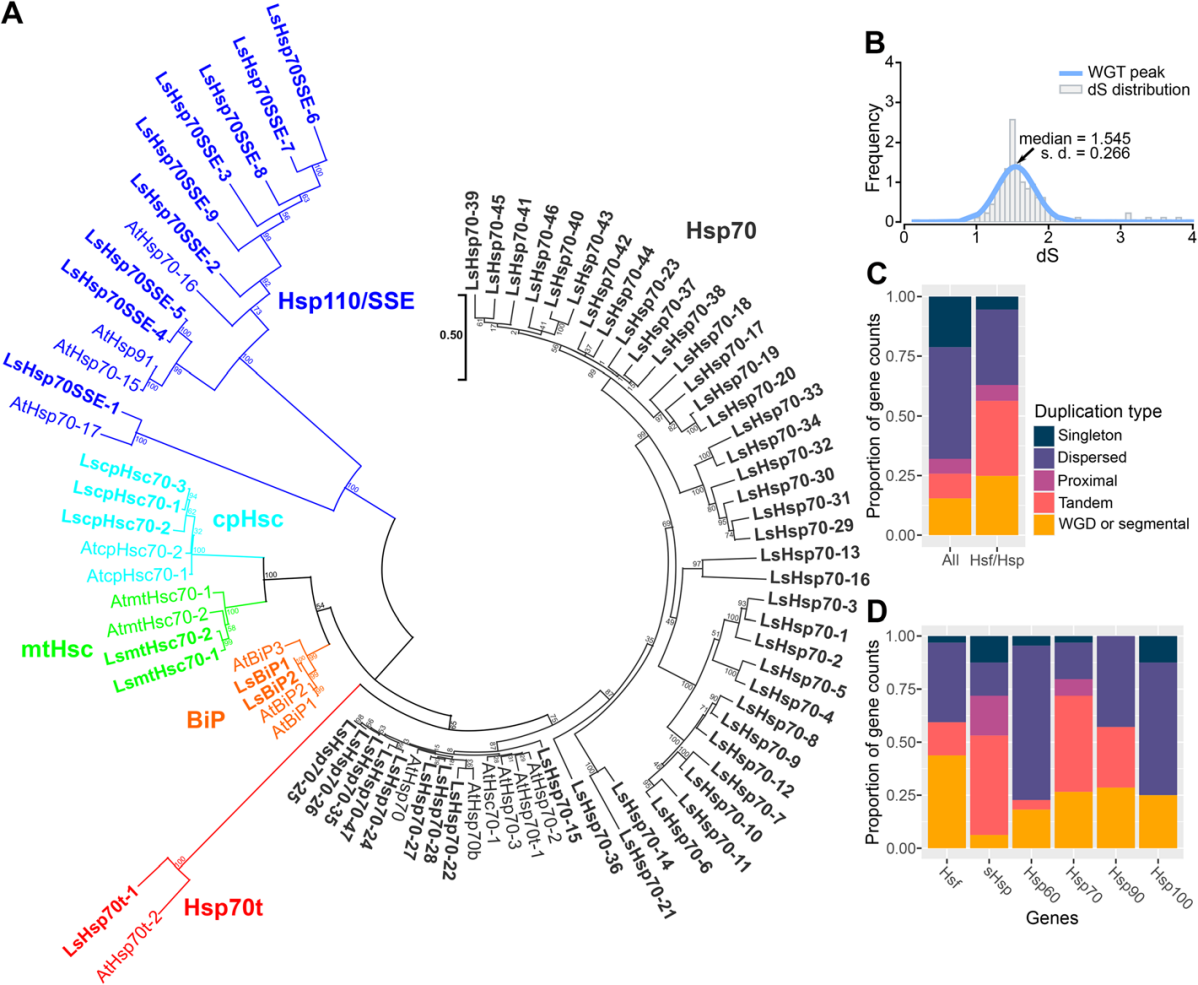
Diversification of lettuce *heat shock protein 70s* (*LsHsp70s*) due to tandem duplication. **a** Phylogenetic relationship of Hsp70 proteins of *L. sativa* and *A. thaliana*. LsHsp70 proteins are marked in bold. *LsHsp70* and *AtHsp70* members in each subfamily (Hsp70, Hsp70t, mtHsc, cpHsc, BiP, and Hsp110/SSE) are represented in different colors. Nomenclature of *LsHsp70* genes was assigned according to the name of *AtHsp70* gene of the highest similarity. **b** Identification of genome triplication events in lettuce. Frequency and distribution of dS values were fitted using Gaussian mixture models. **c** Proportion of singleton, dispersed duplication, proximal duplication, tandem duplication, and whole genome duplication (WGD) or segmental duplication in all lettuce genes and *LsHsf* or *LsHsp* genes. **d** Proportion of different types of duplication in *LsHsf* or *LsHsp* gene families. Phylogenetic analysis was generated by the Maximum Likelihood method. Numbers at the nodes represent percent of bootstrap values based on 500 replications

Hsp70s are molecular regulators of stress responses, as they maintain protein homeostasis by mediating protein folding and/or protein denaturation [29]. Hsp70 family is comprised of DnaK subfamily including cytoplasmic Hsp70 (Hsp70), truncated Hsp70 (Hsp70t), plastidic Hsp70 (cpHsp70), mitochondrial Hsp70 (mtHsp70), endoplasmic reticulum-localized immunoglobin binding protein (BiP), and the Hsp110/SSE subfamily [30]. We found that the Hsp70 subfamily was largely expanded in lettuce (Fig. 5a). We identified nine Hsp110/SSE genes, one Hsp70t gene, three cpHsc genes, two mtHsc genes, and two BiP genes in lettuce (Fig. 5a). Strikingly, the lettuce genome contained 47 cytosolic Hsp70 genes (Fig. 5a), which greatly increased the number of the Hsp70 family genes (64 genes) compared with 18 genes found in Arabidopsis (Fig. 1a). From the 47 members of the cytosolic Hsp70, only five of them have orthologs in Arabidopsis, the remaining 44 members are novel lettuce Hsp60s (Fig. 5a). Another LsHsp70 subclass in which we identified new members was the LsHsp110/SSE (Fig. 5a). In Arabidopsis, four members of the Hsp110/SSE have been described. Our analysis yielded nine members, in which five are clustered together and do not have orthologs in Arabidopsis (Fig. 5a).

A syntenic analysis of lettuce against other plant species of the Asterid order suggested that a whole-genome triplication event occurred in lettuce since its divergence from the grape lineage [2]. To test whether LsHsps and LsHsfs have arisen during whole genome triplication, we identified the dS peak of the lettuce whole genome triplication and estimated the mean dS values across all Hsps and Hsfs paralogs in the lettuce genome (Additional file 5: Table S5). The dS distribution results were fitted using a Gaussian mixture model (Fig. 5b). Interestingly, our analysis showed that lettuce genome triplication did not contribute to the increased gene number of LsHsp70s genes or LsHsfs based on the estimated dS values. Most LsHsfs and LsHsps fall outside of the whole genome triplication peak (mean = 1.545), which support the evidence that the massive increased number of genes within this families arose from a different pattern of duplication. To test what other possible patterns of duplication contributed to the expansion of the LsHsp70s and LsHsfs, we analyzed and compared the patterns of gene duplication across the lettuce genome (Fig. 5c). These pattern of duplications can be classified as dispersed (unpredictable and random patterns distributed across the genome), singleton (reversion to single copy), tandem (closely adjacent genes to each other in the same chromosome), proximal (gene copies that are near each other but separated by several other genes) and whole genome duplication (additional copies of the entire genome are generated) [31, 32]. Dispersed gene duplication (47%) was responsible for the largest number of gene duplications among all gene families within the lettuce genome. Singleton accounted for 21%, followed by whole-genome duplication (16%), tandem duplication (10%) and proximal duplication (6%). When analyzing only Hsfs and Hsps (Fig. 5c), tandem duplication (32%) was the largest responsible for the increased number of genes in both gene families. Dispersed (31%) and whole-genome (15%) duplication were the other two biggest contributors of enlarged gene numbers of Hsfs and Hsps (Fig. 5c).

To further understand what caused the expansion of LsHsp70s, we dissected their type of gene duplication (Fig. 5d). The increased number in LsHsp70s derived largely from tandem duplications, 45% of the total gene duplication types; the large majority were members of the cytosolic LsHsp70s (Additional file 6: Table S6). All the other additional patterns of duplications including singleton, dispersed, proximal and whole genome duplication represented small fractions of the duplication patterns (Fig. 5d). In general, the number of lettuce Hsp70 genes increased more than 3-fold when compared to Arabidopsis and poplar and more than 2-fold compared to rice. Our results support the evidence that an increased number of LsHsp70s is the result of tandem duplication and it is not related to the whole lettuce genome triplication.

**Fig. 6:**
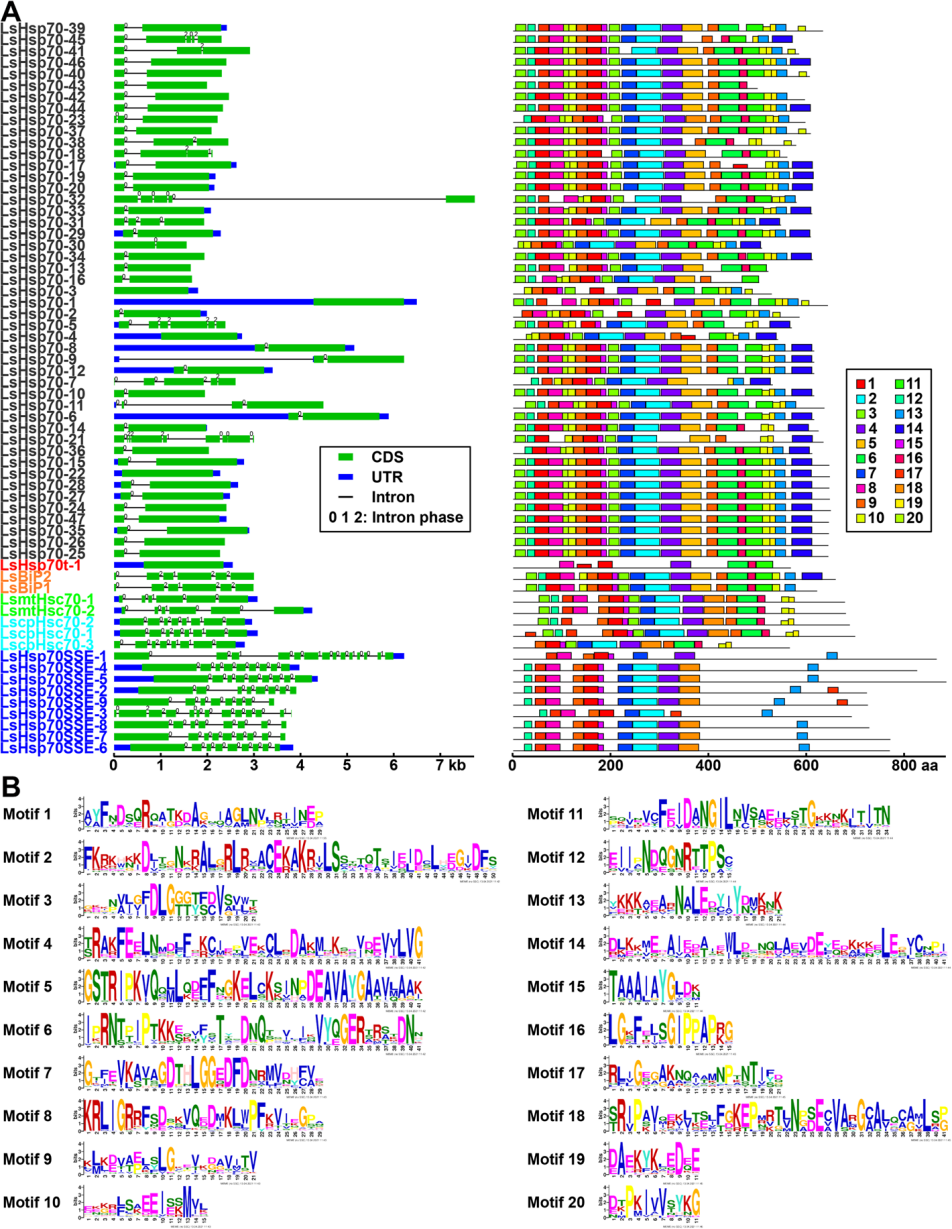
Gene structure and motif composition analysis of *LsHsp70* genes. **a** Blue boxes, green boxes, and black lines indicate untranslated region (UTR), coding sequence (CDS), and intron, respectively. The numbers on each intron represent the intron phase (0, 1, or 2). **b** Conserved motif analysis of LsHsp70 proteins; 20 identified motifs are shown. Phylogenetic analysis was generated by the Maximum Likelihood method. Numbers at the nodes represent percent of bootstrap values based on 500 replications. Detailed information of all motifs is shown in Additional file 4: Table S4

In addition, an interesting gene feature of the Hsp70 subfamily is the low number of exons and the well conserved motif organization. The LsHsp70t subclass single member has no introns and a few motifs, different from all the other subclasses. Cytosolic Hsp70 genes have one intron and a well conserved motif distribution (Fig. 6a). However, some of the residues are not well conserved, except for motifs 5, 7, 8, 15, 16, 19, and 20 (Fig. 6b). Taken together, our results show a large increase in gene number in the LsHsp70 family, which could be related to stress responsiveness to environmental signals.

### Heat shock proteins 90 and 100

Hsp90s and Hsp100s are the two heat shock protein subfamilies with the highest molecular weight (Fig. 1b). Unlike the other LsHsps families, both LsHsp90 and LsHsp100/ClpB families did not increase in gene number during the lettuce whole-genome triplication in comparison with Arabidopsis, rice, and poplar (Fig. 1a). Remarkably, 62% of the LsHsp100s were localized in chloroplasts, 26% in cytoplasm, and 12% in the mitochondria (Fig. 1b). LsHsp90s showed a more diverse localization in several subcellular compartments including ER, nucleus, cytoplasm and chloroplast as well as physiochemical features including MW, pI, and hydropathicity (Fig. 1b; Additional file 1: Table S1).

**Fig. 7:**
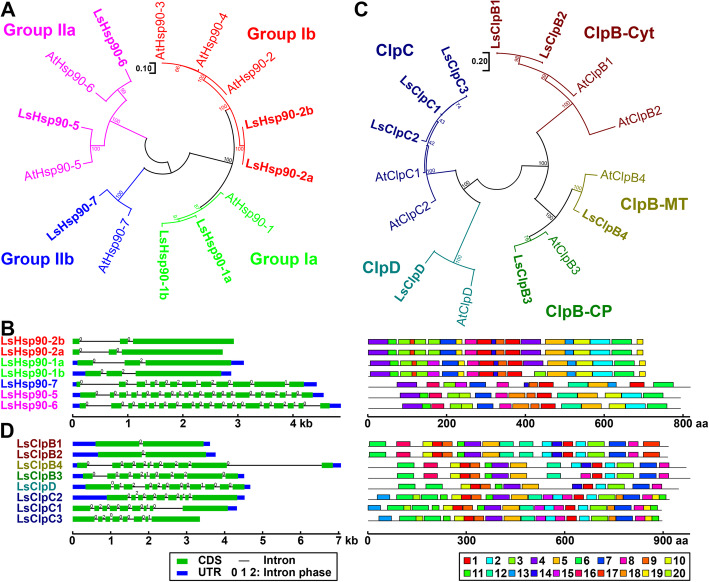
Characterization of lettuce *heat shock protein 90* (*LsHsp90*) and *heat shock protein 100* (*LsHsp100*) gene families. **a** Phylogenetic tree of Hsp90 proteins of *L. sativa* and *A. thaliana*. Lettuce Hsp proteins are marked in bold. **b** Gene structure and motif composition analysis of *LsHsp90.* **c** Phylogenetic analysis of Hsp100 proteins of *L. sativa* and *A. thaliana.* **d** Gene structure and motif composition analysis of *LsHsp100.* **a, c** Nomenclature of *LsHsp90* and *LsHsp100* genes were assigned according to the name of *A. thaliana* homologues*.* Lettuce *Hsp90* and *Hsp100* family members are represented in different colors according to their subfamily (Group Ia, Ib, IIa, and IIb in Hsp90ClpB-Cyt, ClpB-MT, ClpB-CP, ClpC, and ClpD in Hsp100. **c, d** Exon-intron organization. Blue boxes, green boxes, and black lines indicate untranslated region (UTR), coding sequence (CDS), and intron, respectively. The numbers on each intron represent the intron phase (0, 1, or 2). Phylogenetic analysis was generated by the Maximum Likelihood method. Numbers at the nodes represent percent of bootstrap values based on 500 replications. Detailed information of all motifs is shown in Additional file 4: Table S4

Plant Hsp90 family proteins have been classified into two main groups (group I and II), and are divided into four subclasses (groups Ia, Ib, IIa, and IIb) [33]. Similarly, LsHsp90s subfamily contains seven members, organized in four subclasses. Group Ia includes LsHsp90-1a and LsHsp90-1b (Fig. 7a), both orthologous genes of a single member described in Arabidopsis. Similarly, group Ib contains only two members orthologs of AtHsp90–2/3/4 group (Fig. 7a). At the gene structure level, LsHsp90s can be divided in two main groups based on their exon and motif numbers (Fig. 7b). All the members of the group Ia and Ib have three exons and a highly similar motif structure. In contrast, members of the group IIa and IIb have a larger number of exons (19 and 12 exons, respectively) and a different motif pattern compared to the group Ia and Ib, but a similar motif pattern similar among them (Fig. 7b).

LsHsp100/ClpB family contains five subclasses (Fig. 7c), CplB-Cyt (cytoplastic localized), CplB-CP (chloroplast localized), ClpB-MT (mitochondrial localized), ClpC, and ClpD. Phylogenetic analysis shows that lettuce and Arabidopsis shared a comparable number of group members with similar localization (Fig. 7c). Lettuce Hsp100 family genes also showed structural similarities depending on their subclasses. Cytoplasmic ClpB genes included only two exons; however, chloroplastic and mitochondrial ClpB, ClpC, and ClpD genes contained between eight to twelve exons (Fig. 7d).

### Chromosomal distribution and duplication events of Hsf and Hsp genes in lettuce

**Fig. 8:**
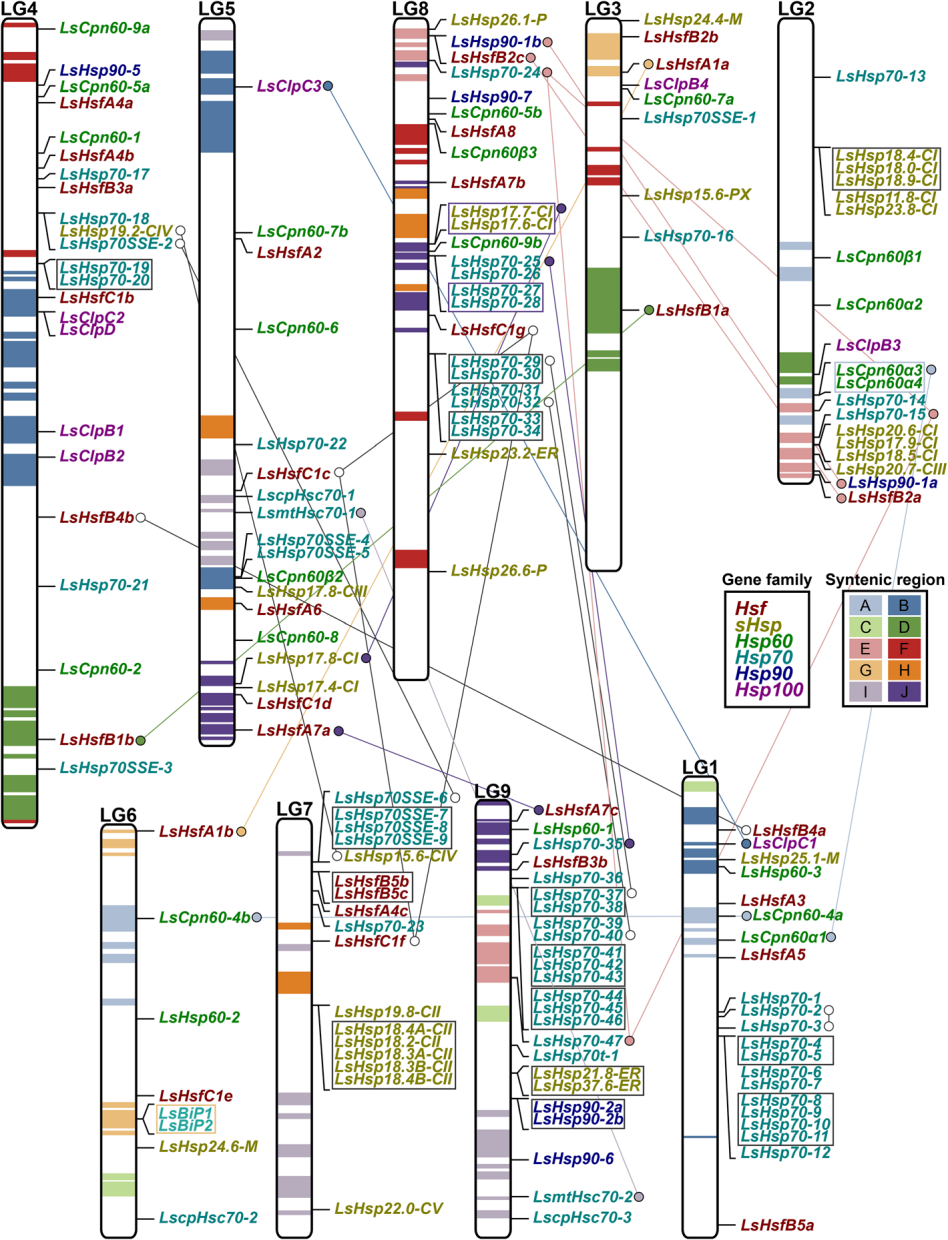
Genome-wide distribution of tandem duplicated regions highlighting chromosomal location of Hsfs and Hsps genes. Lettuce syntenic regions are shown with colored blocks in each chromosome (LG1 to LG9). Physical location of each LsHsf or LsHsp gene is shown and gene names are colored according to their gene family. LsHsfC1a (Lsat_1_v5_gn_0_11820) was not included because it has not been mapped on any of the nine chromosomes in lettuce. Boxes represent tandemly duplicated genes. Paralogous gene pairs originated from segmental duplications are highlighted with circles and connected by lines

In our initial analysis, we found an increased number of Hsfs and Hsps in lettuce compared to other species (Fig. 1a). To further investigate which particular Hsfs and Hsps subfamilies underwent duplication or genome lost, we first analyzed their chromosomal distribution. A total combined of 165 Hsfs and Hsps were randomly distributed in each of the nine lettuce chromosomes (Fig. 8). Using syntenic regions previously categorized [2], we searched for Hsps and Hsfs located in those regions (Fig. 8). Remarkably, 43.7% (14 out of 32) of the LsHsfs were located in syntenic regions. Syntenic region A was found in chr1, chr2, and chr6 and only contained Hsps subfamily 60, in particular subclass LsCpn60. Syntenic region B is present in chr1, chr4 and chr5 and carries mostly members of the LsClp subclass (LsHsp100). Syntenic region D carries LsHsfB1a in chr3 and LsHsfB1b in chr4. It was also present in chr2; however, no other LsHsf was located in that syntenic region (D). Syntenic region E was more heterogenous and it is present in chr2, chr8, and chr9. It carries LsHsfs and members of the LsHsp70 and LsHsp90. Interestingly, syntenic region G was only located in chr3 carrying LsHsfA1A and LsHsfB2b, while chr6 carries LsBiP1 and LsBiP2 (LsHsp70). Syntenic region I contains LscpHsc70–1 and LsmtHsc70–1 in chr5 and their duplicated genes LscpHsc70–3 and LsmtHsc70–2 were found in chr9 respectively. Syntenic region H carries a diverse mix of LsHsfs and LsHsps and it is located in chr5, chr8 and chr9. Finally, syntenic regions C, F and H do not carry any Hsfs and Hsps.

To further elucidate whether LsHsps were localized as gene clusters within the lettuce genome, we performed a collinearity analysis within the lettuce genome using the Multiple Collinearity Scan toolkit X version (MCScanX) with an E-value of 10^− 5^ (Additional file 5: Table S6). The large majority of gene clusters found in the lettuce genome represent genes within the LsHsp70s subfamily (Fig. 8), and are distributed in chr1, chr4, chr7, chr8, and chr9. Interestingly, chr1 and chr9 carry gene clusters with six and eight LsHsp70s genes, respectively. This indicates that the greater number of Hsp70 genes found in lettuce compared to Arabidopsis, rice, and poplar could be the result of clustered tandem duplication. Similarly, chr8 carries two gene clusters of LsHsp70s. A second group of abundant gene clusters found in the lettuce genome belongs to the LssHsps family. Tandem repeat of LssHsps gene clusters were found in chr2, chr7, chr8, and chr9. Our results showed that the increased number of LsHsfs and LsHsp70s that we found in the lettuce genome were the result of tandem duplications.

### Cis-element analysis of lettuce Hsf and Hsp genes

**Fig. 9:**
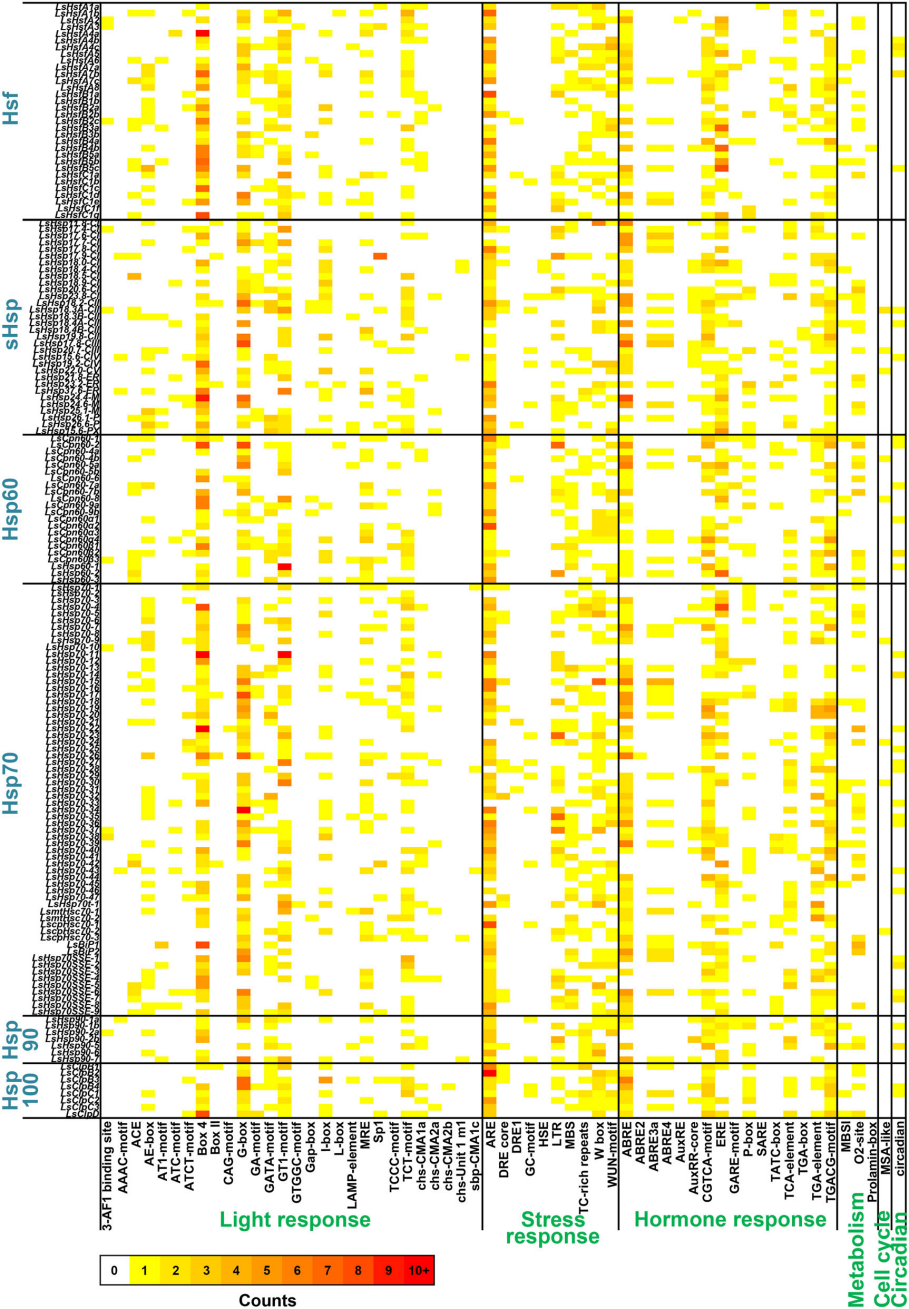
Cis-regulatory element analysis of lettuce *Hsf* and *Hsp* genes. Putative cis-regulatory elements were analyzed in the 2 kb upstream regions of *LsHsf* and *LsHsp* genes. Six groups of cis-regulatory elements (light response, stress response, hormone response, metabolism, cell cycle, and circadian rhythm) are displayed. Colors from white to red indicate the occurrence of each cis-regulatory element from 0 to 10+ (10 or more)

In order to understand how LsHsfs and LsHsps are regulated, we performed a Cis-regulatory element analysis of LsHsfs and LsHsps promoters using a 2 kb upstream region of each gene. We particularly focused on Cis-regulatory elements related to light, stress, and hormone responses as well as metabolism, cell cycle, and circadian rhythm (Fig. 9; Additional File 6: Table S7). A total number of 59 cis-regulatory elements were identified among LsHsfs and LsHsp genes. Interestingly, within the light responsive cis-elements, G-box, GT1 motif, TCT motif, and box 4 were highly abundant in all LsHsf and LsHsp gene families. ARE cis-element was the most abundant in the stress responsive category, having multiple ARE motif present across the promoter of both LsHsfs and LsHsps genes (Fig. 9; Additional File 6: Table S7). In the hormone stress category, ABRE was ubiquitously present in the LsHsfs promoters and in most of the LsHsp promoters. However, ABRE motif was underrepresented in promoter regions of LsHsp90s (Fig. 9; Additional File 6: Table S7). Cis-elements related to metabolism, cell cycle and circadian rhythm were less abundant and indicate the low involvement of LsHsfs and LsHsps in these biological processes.

### Transcriptional analysis of LsHsfs and LsHsps under different light conditions

**Fig. 10:**
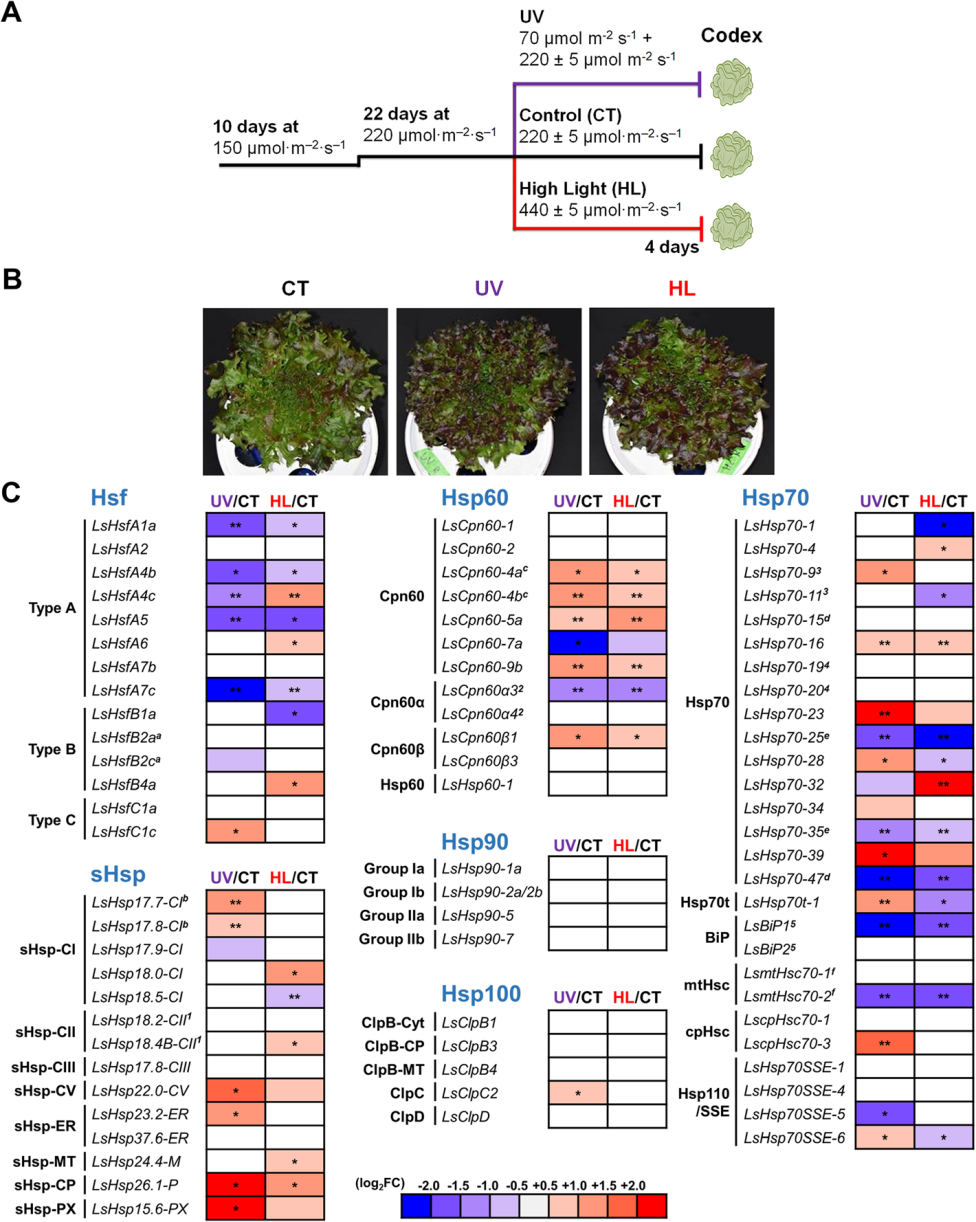
Gene expression analysis of lettuce *Hsf* and *Hsp* genes under different light conditions. **a** Schematic representation of the experimental design used at different light treatment on lettuce plants. *L. sativa* cultivar Codex plants were grown under light 150 μmol·m^− 2^·s^− 1^ for 10 days then 220 μmol·m^− 2^·s^− 1^ was treated for 22 days. Control light (μmol·m^− 2^·s^− 1^), UV light (11 μmol·m^− 2^·s^− 1^ supplemented with 220 μmol·m^− 2^·s^− 1^ of white light), and high intensity light (440 μmol·m^− 2^·s^− 1^) were imposed to lettuce plants for 4 days. **b** Physical appearance of *L. sativa* cultivar “Codex” 4 days after treatment with control light (CT), UV, and high intensity light (HL). **c** The relative expression of lettuce Hsf and Hsp genes. mRNA levels were determined by RT-qPCR analysis and normalized to that of LsTUB. log_2_FC (fold change) values of relative expression of treatment (UV and HL) to the control (CT) are presented. Asterisks indicate significant differences between treatment and control determined by Wilcoxon’s singed-rank test (* *p* < 0.05, ** *p* < 0.01, *** *p* < 0.001). Genes marked with the same letter (a-f) represent segmentally duplicated genes; genes marked with the same number (1–5) belong to the same tandem duplicated gene cluster

Many studies have shown that Hsfs and Hsps genes are involved in plant responses to abiotic stresses in model plants [12] and other crop species [10, 14, 24, 34]. To investigate the role of LsHsfs and LsHsps under different light conditions, we grew lettuce plants *cv. Codex* in hydroponic conditions. After germination, lettuce plants were grown for 10 days under broad-spectrum LED lamps providing an average of 150 ± 5 μmol·m^− 2^·s^− 1^. Then, light conditions were adjusted to 220 ± 5 μmol·m^− 2^·s^− 1^ for 22 days. Control plants were kept at the same light conditions while UV treated plants were exposed to 220 μmol·m^− 2^·s^− 1^ supplemented with UV radiation. High intensity light condition was achieved by exposing plants to 440 ± 5 μmol·m^− 2^·s^− 1^. All end-of-production treatments were applied for 4 days and then mature lettuce plants were harvested for gene expression analysis (Fig. 10a). In general, lettuce plants under UV and high intensity light conditions became reddish, while lettuce leaves retained a green color under control conditions (Fig. 10b).

We selected representative members of each subfamily of LsHsfs and LsHsps based on our genome-wide analysis. In general, LsHsfs type A members were highly responsive to UV and high intensity light stress. A similar response previously observed in Arabidopsis [16]. LsHsfA1 LsHsfA4b, LsHsfA5 and LsHsfA7c, type A Hsfs, showed significant downregulation when lettuce plants were exposed to UV radiation and high intensity light conditions (Fig. 10c). Interestingly, LsHsfA4c was downregulated in response to UV light but upregulated under high intensity light. In contrast, LsHsfs type B was not responsive to UV light. Only two members of this family showed transcriptional changes; LsHsfB1a was downregulated and LsHsfB4a upregulated in response to high intensity light but did not respond to UV radiation, similarly as all the members analyzed in this type of LsHsfs. In the case of Hsfs type C, LsHsfC1c did not change neither to UV light nor high intensity light stress. LsHsfC1c was upregulated in response only to UV radiation (Fig. 10c).

Remarkably, almost all subclasses of LsHsps were upregulated in response to at least one of the treatments, UV radiation or high intensity light. Members of the LsHsp-CP and LsHsp-PX subclasses together with LsHsp17.7-Cl, LsHsp17.8-Cl, LsHsp22.0-CV and LsHsp23.2-ER were significantly upregulated in response to UV treatment but did not respond to high intensity light (Fig. 10c).

Transcriptional expression of LsHsp60s under UV radiation and high intensity light showed primarily upregulation of its members; as shown by LsCpn60-4a, LsCpn60-4b, LsCpn60-5a, LsCpn60-9b and LsCpn60ß1. However, although LsCpn60–2, LsCpn60α4, LsCpn60ß3, and LsCpn60–1 did not respond to neither of the treatments, LsCpn60-7a and LsCpn60α3 were downregulated for both treatments, UV radiation and high intensity light (Fig. 10c).

In relation to LsHsp70s, most of the novel members identified were responsive to both treatments, UV and high intensity light stress. The transcriptional response of LsHsp70s was variable. LsHsp70–23 and LsHsp70–39 were significantly upregulated by UV light, but did not respond to high intensity light. Oppositely, LsHsp70–32 was upregulated in response to high intensity light stress but did not significantly change under UV conditions (Fig. 10c).

Interestingly, LsHsp70t-1 was significantly upregulated and downregulated under both UV and high intensity light. LsBiP1 and LsmtHsc70–2 were both downregulated under UV and high intensity light. Remarkably, LsHsp90 and LsHsp100 did not respond to UV and high intensity light treatment, suggesting that they might not be involved in responding to light stress.

Since we found tandem duplication of several LsHsps genes, we selected several of those genes to explore the possibility of cluster expression in response to light conditions. Surprisingly, we did not find any transcriptional response of duplicated genes based on our RT-qPCR data (Fig. 10c). Interestingly, LsHsp18.2-CII did not respond to light stress; however, LsHsp18.4b-CII, located in the same duplicated cluster, was significantly upregulated in response to high intensity light. Another example was the case of LsCpn60α3 and LsCpn60α4 in which the former was significantly downregulated in response to both light treatments, but the latter did not show changes in gene expression (Fig. 10c). Because most of the new LsHsp70s members that we identified are located in clusters (Fig. 8), we tested three of these clusters containing LsHsp70s genes (Fig. 10c). We found that none of the genes tested show similar transcriptional pattern. It is worth noticing that functional characterization of those clusters might provide a better understanding of their regulatory role.

## Discussion

Plant stress responses are frequently the result of simultaneous mechanisms that synergistically and/or coordinately operate to prevent, maintain, and re-establish cellular homeostasis [35–37]. One of the best characterized stress mechanisms is the heat shock proteins response. It has been demonstrated that Hsps play an important role in many cellular processes in response to stressful conditions, which may disclose whole plant responses to multiple environmental stresses. This particular stress response mechanism involves the activation of different Hsp families [29, 34] regulated by Hsfs to produce a rapid and a constitutive response. Thus, the different classes of Hsps work together with Hsf to maintain cellular homeostasis [12, 29]. In this study, we performed a genome-wide analysis for Hsfs and Hsps in lettuce and identified an overall increase in gene number in most of these families.

### Diversification of LsHsfs

The composition of the Hsfs family has so far been fully described in model plant species as well as in some cereal crops [10, 12, 14, 15, 38]. There are 21 Hsfs in Arabidopsis (*Arabidopsis thaliana*), 27 Hsfs in tomato (*Solanum lycopersicum*), 28 in rice (*Oryza sativa*), 31 in maize (*Zea mais*), and 25 in poplar (*Populus trichocarpa*) [23, 38–40]. However, several studies have shown that Hsfs is subject to evolutionary changes based on both number and composition of the Hsf family [40]. Our genome-wide analysis in lettuce identified 32 Hsfs genes; a higher number compared to Arabidopsis (Fig. 1a; Fig. 2a). This increase in gene number resulted largely because of tandem duplication in the lettuce genome [2]. Interestingly, unlike Hsps, almost all Hsfs are not located in tandem repeats in the lettuce genome, with the exception of LsHsf5b and LsHsf5c which are positioned in tandem repeats in chr7 (Fig. 8). This suggests that all LsHsfs are functional genes with a possible role in the stress response and homeostasis maintenance.

The basic structure of Hsf proteins is formed by a DNA binding domain, oligomerization domain, and a C-terminal activation domain. Classification of plant Hsfs (A, B, and C) is mainly based on variances in these three domains, particularly the oligomerization domain. The most evident difference is the insertion of additional amino acid residues in the oligomerization domain of type A and type C Hsfs as well as the absence of transcriptional activator motifs in the C-terminal domain of type B and C Hsfs [10, 38]. In Lettuce, all the Hsfs follow the same basic structure. For instance, the type C members lack C-terminal domains resulting in smaller number of genes (Fig. 2b). Type A Hsfs are known to function as transcriptional activators of stress genes. Genetic and functional analysis suggests that HsfA1a and HsfA1b are central regulators required in the early phase of the heat shock response [35, 37, 41]. In Arabidopsis, members of the Hsfs type A function as the main positive regulators in heat shock-responsive gene expression including members of the Hsp70 and Hsp90 families [42, 43]. We observed a strong downregulation of LsHsfA1a under UV and high intensity light (Fig. 10), which might explain the lack of response of LsHsp90s and LsHsps100s to stress conditions under UV and high intensity light.

### Tandem duplication played a major role in the diversification of the LsHsp70 family

In plants, gene duplication through whole-genome duplication (WGD), singleton, tandem, proximal or segmental events represent a major force driving gene family expansion [44, 45]. Similarly as described in many other plant species [12, 14], lettuce Hsfs and Hsps have experienced complex biological rearrangements resulting in adaptation to specific conditions (Fig. 8) [2]. Our phylogenetic analysis comparing LsHsp70s with Arabidopsis, rice, and poplar revealed six different groups, designated as cytosolic Hsp70, Hsp70t, BiP, MtHsc, cpHsc and Hsp110/SSE (Fig. 5a). We found that the LsHsp70 family was the one that had undergone the largest tandem duplication within the Hsps. Out of 64 LsHsp70 gene members 39% (25 members) are localized in duplicated blocks in the lettuce genome.

Interestingly, Hsp70s have undergone tandem duplication in animals [46, 47], mosses [48] and plant species [49, 50]. For instance, in potato (*Solanum tuberosum*) [49] and moss (*Physcomitrella patens*) [48], tandem and segmental duplication events contributed to the expansion of the Hsp70 members in both species. Due to the nature of the different organisms in which the expansion of the Hsp70 family have been found as well as the variety of stress responses, Hsp70 genes are described as vital genetic elements in response to abiotic stresses. However, it is important to notice that even though Hsps underwent tandem duplication in potato and moss, the expansion was not as massive as the one we found in lettuce. Our findings suggest that LsHsp70 suffered dynamic rearrangements that allowed the emergence of novel members of the LsHsp70 family (Fig. 5) which was largely due to tandem duplications. Interestingly, 39% of the LsHsp70s have a single exon, all of them from the highly abundant cytosolic Hsp70 subclass (Fig. 6), which is in line with previous studies which suggest that the evolution of several plant gene families is associated with the diversification of exons and introns within those gene family members [51].

### Light quality effects on LsHsfs and LsHsps transcriptional levels

During a plant’s life cycle, a large number of essential processes are regulated by light. These processes include photosynthesis, plant growth and development, anthocyanin biosynthesis, and gene expression, among others [3, 4, 52, 53]. In Arabidopsis, three A-type Hsfs, HsfA1D, HsfA2, and HsfA3 were found to regulate early responses during excess light energy [16]. In lettuce, under UV and high intensity light, we found strong downregulation of LsHsfA1a and LsHsfA7c (Fig. 10c). In addition, LsHsfB1a was downregulated in response to high intensity light and LsHsfC1c upregulated in response to UV.

Interestingly, two genes located in the same clade, LsHsp18.4B-CII and AtHsp17.6-CI (Fig. 3a) showed similar transcriptional expression patterns in response to high intensity light and UV exposure (Fig. 10c). In lettuce, LsHsp18.4B-CII was upregulated by high intensity light but did not respond to UV light. Similarly in Arabidopsis, AtHsp17.6-CI was also induced by high intensity light treatment, however, no change in transcript abundance was detected under UV light [54]. The similarity of expression patterns between small heat shock proteins in lettuce and Arabidopsis might indicate a cohesive family-level expression pattern in response to environmental stresses as previously shown in Arabidopsis [12].

An interesting finding was the fact that under different light conditions, most studied members of the Lettuce Hsp90 and Hsp100 families did not change transcriptionally. In contrast, most members of the LssHsp and LsHsp60 families were significantly up-regulated (Fig. 10c). In general, Hsp90 and Hsp100 are stress-regulated by many abiotic and biotic stresses [55], however, in Arabidopsis, gene members of Hsp90s and Hsp100s showed no expression or slight changes across stress conditions, including UV [12]. While further studies need to be conducted to elucidate the functions of these genes under UV and high intensity light conditions, our data suggests that the hydroponic conditions we used in this study are not able to trigger molecular responses in LsHsp90s and LsHsp100s, similarly as observed in Arabidopsis.

The specific response of many members of the LsHsp70 family to UV and high intensity light might be associated with the increased member duplication of the cytosolic Hsp70 in lettuce. Under excessive light energy, damage of the electron transport chain as resulting in irreversible impairments of several subunits of the photosystem II (PSII) has been observed [56–58]. Thus, plants first try to dissipate excess light stored as electron energy in the chloroplasts to avoid heat damage [56]. In the photosynthetic green algae *Chlamydomonas reinhardtii,* overexpression of HSP70 conferred photoprotection and repair of PSII during and after photoinhibition, whereas decreased levels (antisense construct) of Hsp70 caused an increased light sensitivity [58]. Similarly as observed in lettuce, the increased number of Hsp70 members might be associated with enhanced capability to withstand high intensity light as well as UV damage.

Under UV conditions, we observed a high transcriptional expression of Hsfs and Hsps. For instance, a strong downregulation of LsHsp70–28 and LsHsp70t-1 especially due to high intensity light conditions, but a significant upregulation of the aforementioned Hsps under UV (Fig. 10c) was observed. It is well documented that UV exposure induces generation of reactive oxygen species [59]. While reactive oxygen species are important signaling molecules, when highly produced they cause severe damage to plant cells [60]. Hsp70s regulate cellular redox status by maintaining the levels of reactive oxygen species [55]. Analysis of an Arabidopsis cytosolic Hsp70 protein sequence showed a high number Cys residues compared with other Hsps [54]. Cys residues are key elements in the redox regulation. Thus, the high transcriptional level of several of the lettuce Hsp70s under UV light might be associated with the maintenance of the redox status. Our study provides initial assessment of lettuce Hsfs and Hsps under different light conditions; however, the precise regulatory mechanisms of lettuce grown hydroponically under control and stress conditions require further investigation.

## Conclusions

Our detailed genome-wide analysis on the heat shock factors and heat shock proteins in lettuce identified 32 and 165 genes, respectively. Phylogenic analysis of Hsfs and Hsps genes highlight a close relationship with their orthologous genes in Arabidopsis. Interestingly, a large number of novel members were also found in Hsfs, sHsps and Hsp70 gene families as a result of the tandem duplication. Several Hsfs, sHsps, Hsp60s, and Hsp70s genes are highly responsive to UV and high intensity light conditions and provide candidates for breeding programs aiming to produce lettuce varieties able to grow healthier under hydroponic systems that use artificial light.

## Methods

### Plant material and growth conditions

Lettuce seeds of red-leaf cultivar cv. *Codex rz* were pre-germinated for 48 h until radicle emergence was observed. Germinated seeds were transplanted into rockwool plugs [3]. Seedlings were propagated for 10 days inside a walk-in growth chamber using an average photosynthetic photon flux of 150 ± 5 μmol·m^− 2^·s^− 1^ (24-h photoperiod) and a daily light integral (DLI) of 12.96 mol·m^− 2^·d^− 1^ provided by broad-spectrum LED lamps (Philips GP150 Red/Deep Red/Blue Low Blue High Output). Temperature and relative humidity (RH) were set at 24 °C and 60 to 80%, respectively; CO_2_ concentration was maintained at ambient levels. Seedlings of similar size were transplanted into individual deep-water culture closed hydroponic systems using plastic cups with a 5 cm diameter. Plants were grown using a commercial water-soluble fertilizer (OASIS® Grower Solutions Hydroponic Fertilizer 16–4-17) dissolved in tap water at a concentration of 150 mg·L^− 1^ N (EC and pH = approx. 1.2 dS·m^− 1^ and 6.0, respectively). Each 7.6 L hydroponic system had a clear plastic tube attached to an air pump to provide continuous aeration.

### Light treatments

Lettuce plants were grown inside a growth chamber equipped with two opposite multi-level shelves. Each shelf contained a block with four levels used as treatment compartments. All compartments held four hydroponic systems. Plants were grown under broad-spectrum LED lamps providing an average DLI of 15.84 mol·m^− 2^·d^− 1^ (220 ± 5 μmol·m^− 2^·s^− 1^; 20-h photoperiod from 02:00 to 22:00 h). The average ambient day (from 02:00 to 22:00 h) and night (from 22:00 to 02:00 h) air temperature of the chamber was set at 22 °C and 21 °C, respectively; CO_2_ concentration, and RH was set at 405 ppm and 60 to 80%, respectively. After 22 days in the chamber, plants were subjected to one of three treatments during 4 days: end-of-production (EOP) UV-A (11 μmol·m^− 2^·s^− 1^) + white light (220 μmol m^− 2^·s^− 1^), EOP high intensity light (440 ± 5 μmol·m^− 2^·s^− 1^) and a control (220 ± 5 μmol·m^− 2^·s^− 1^) with no EOP treatment (Fig. 10a).

### Sequence retrieval and phylogenetic reconstruction

Heat Shock Factors (Hsfs) and Heat Shock Proteins (Hsps) sequences of Arabidopsis, rice, and poplar [12, 14, 15] were retrieved from public databases. When a gene had splice variants, primary transcripts were selected as representative for the gene. To identify putative lettuce *Hsfs* and *Hsps,* retrieved sequences were used as queries using an automated BLASTP search against the lettuce genome database in Phytozome [61]. An E-value threshold of 1·E^− 20^ was used. When multiple transcripts were predicted for a locus from BLASTP, a transcript with the lowest E-value was chosen as a representative for the locus. To construct orthologous gene families, the OrthoFinder tool was used [26].

Phylogenetic trees were generated by the Maximum Likelihood method and Le_Gascuel_2008 model [62] with 500 Bootstrap replications using MEGA X software [63] as previously described [64]. A discrete Gamma distribution was used to model evolutionary rate differences among sites. Gene names of lettuce Hsfs and Hsps were assigned based on their phylogenetic relationship with Arabidopsis proteins. Lettuce sHsps were classified according to their molecular weights obtained using the ProtParam tool [65]. Phylogenetic analysis data was deposited in the repository of phylogenetic information, TreeBASE and it is available in the following link: http://purl.org/phylo/treebase/phylows/study/TB2:S27434?x-access-code=f2368699e8e38ce6d23cad64f44dc452&format=html

### Physical and chemical properties of heat shock factors and heat shock proteins

The physical and chemical properties including molecular weight, isoelectric point, instability index, aliphatic index, and hydropathicity of all lettuce Hsfs and Hsps were estimated using ProtParam tool from ExPASy server [65]. Protein subcellular localization of lettuce Hsfs and Hsps were predicted using WoLF PSORT [66]. Multiple sequence alignment for Arabidopsis and lettuce Hsfs and Hsps were performed by MUSCLE [67].

### Chromosomal mapping and collinearity analysis of Hsf and Hsp genes in the lettuce genome

The chromosomal locations of Lettuce Hsfs and Hsps genes were retrieved from Phytozome, *Lactuca sativa V5* genome database [61]. All lettuce protein sequences were included in a local database using an automated Basic Local Alignment Search Tool (BLAST). The BLASTP results were analyzed by the Multiple Collinearity Scan toolkit X version (MCScanX) [68] using an E-value of 10^− 5^ to produce collinearity blocks across the whole genome. The collinearity pairs belonging to Hsfs and Hsps were extracted to draw a collinearity map.

### Non-synonymous (dN) to synonymous substitution (dS) ratio (dN/dS) analysis

The Ratio between non-synonymous mutations (dN) to synonymous mutation (dS) of each lettuce Hsf or Hsp gene and their corresponding Arabidopsis ortholog was calculated using EMBOSS Water pairwise alignment [69]. The dN/dS ratio was estimated by utilizing the PAL2NAL tool with the input of the pairwise DNA sequence alignment [70].

To analyze the whole genome triplication peak (dS), we used the γ-MYN method [71] to calculate dN and dS values by implementing the Tamura–Nei model as previously described [32]. The dS values > 4 were excluded from further analysis due to the saturated substitutions at synonymous sites. The dS distribution of lettuce whole genome triplication pairs was fitted using the Gaussian Mixture Model.

### Gene structure, conserved motif and Cis-regulatory element analysis of LsHsf and LsHsp genes

Exon-intron structures of lettuce Hsfs and Hsps were depicted using the Gene Structure Display Server – GSDS v2.0 [72]. Genomic sequence and coding sequences of each Hsf or Hsp gene were downloaded from Phytozome database and aligned to predict the exon-intron structure.

Conserved motifs analysis of Hsfs and each Hsp family were determined using Multiple Em for Motif Elicitation (MEME) Suite online program with the following parameters: maximum motif numbers = 20; site distributions = any number of repetitions; motif width = 6 to 50 [73].

Cis-regulatory elements of Lettuce Hsf and Hsp genes were identified using 2000 bp upstream regions of each lettuce Hsf and Hsp genes using the PlantCARE database [74].

### RNA isolation and RT-qPCR

Total RNA from leaves of twenty-two days old mature plants was isolated using Trizol (Ambion) following the manufacturer’s instructions. cDNA synthesis was performed using reverse transcription system (Invitrogen SuperScript II) and oligo (dT) primers. Real-time PCR reactions were performed using SYBR Green FastMix (Quantabio) as previously described [75]. Lettuce Tubulin (LsTUB) was used as housekeeping gene for internal normalization control. RT-qPCRs were performed in the QuantStudio™ 3 Real-Time PCR System (Applied Biosystems) in a 96-well reaction plate. Primers used are described in Additional File 7: Table S8. Cycling parameters consisted of 5 min at 95 °C, and 45 cycles of 95 °C for 15 s, 60 °C for 30 s, and 72 °C for 30 s [76]. RT-qPCR reactions were performed in triplicate for each RNA sample on three biological replicates of each light condition. Specificity of the amplifications was verified by a melting curve analysis. Relative amounts of mRNA were calculated from threshold points (Ct values) located in the log-linear range of real-time PCR amplification plots using the 2^-ΔΔCt^ method [77].

### Statistical analysis

R software/environment was used for the statistical analyses of the RT-qPCR data. Three independent experiments in which each experiment had four biological replicates were used. Wilcoxon’s singed-rank test [78] was used to compare gene expression between the experimental groups (UV and high intensity light) and the control group (normal light). Differences in means were considered significant at *p*-value < 0.05.

## Supplementary Information

**Additional file 1: Table S1.** List of *LsHsf* and *LsHsp* genes and their physicochemical properties.

**Additional file 2: Table S2.** dNdS ratio between Lettuce genes and Arabidopsis homologs.

**Additional file 3: Table S3.** Orthogroups among Hsfs and Hsps genes in *A. thaliana* and *L. sativa.*

**Additional file 4: Table S4.** Multiple EM for Motif Elicitation (MEME) analysis of each gene family.

**Additional file 5: Table S5.** dS peaks calculated using the γ-MYN method.

**Additional file 6: Table S6.** Tandem and segmental duplication of lettuce *Hsf* and *Hsp* genes.

**Additional file 7: Table S7.** Cis-regulatory element analysis present in 2-kb promoter regions of *LsHsf* and *LsHsp* genes.

**Additional file 8: Table S8.** List of primers used in RT-qPCR analysis.

**Additional file 9.** Supplementary Index.

## Data Availability

All data generated or analyzed during this study are included in this published article and its supplementary information files.
