# Supplementary material for: Genome-wide identification of heat shock factors and heat shock proteins in response to UV and high intensity light stress in lettuce
Source: BMC Plant Biol. 2021 Apr 17;21:185. doi: 10.1186/s12870-021-02959-x (PMC8053295; doi:10.1186/s12870-021-02959-x)
Supplement: Supplementary file 9 — Additional file 9. Supplementary Index. [file 12870_2021_2959_MOESM9_ESM.docx]

**Title:** Genome-wide identification of Heat shock factors and Heat shock proteins in response to UV and high intensity light stress in lettuce.

Taehoon Kim^1^, Shafina Samraj^1^, Juan Jimenez^1^, Celina Gómez^1^, Tie Liu^2^, Kevin Begcy^1*^

^1^University of Florida, Environmental Horticulture Department, Gainesville, Florida 32611

^2^University of Florida, Horticultural Science Department, Gainesville, Florida 32611

Additional Table S1. List of *LsHsf* and *LsHsp* genes and their physicochemical properties.

Additional Table S2. dNdS ratio between Lettuce genes and Arabidopsis homologs.

Additional Table S3. Orthogroups among Hsfs and Hsps genes in *A. thaliana* and *L. sativa.*

Additional Table S4. Multiple EM for Motif Elicitation (MEME) analysis of each gene family.

Additional Table S5. dS peaks calculated using the γ-MYN method.

Additional Table S6. Tandem and segmental duplication of lettuce *Hsf* and *Hsp* genes.

Additional Table S7. Cis-regulatory element analysis present in 2-kb promoter regions of *LsHsf* and *LsHsp* genes.

Additional Table S8. List of primers used in RT-qPCR analysis.
